# Sculpting new structures

**DOI:** 10.7554/eLife.57668

**Published:** 2020-05-28

**Authors:** Jocelyn A McDonald, Yoshinori Tomoyasu

**Affiliations:** 1 Division of Biology, Kansas State UniversityManhattanUnited States; 2 Department of Biology, Miami UniversityOxfordUnited States

**Keywords:** developmental evolution, genitalia, apical extracellular matrix, morphogenesis, *Drosophila biarmipes*, *D. melanogaster*

## Abstract

The origins of the posterior lobe, a recently evolved structure in some species of *Drosophila*, have become clearer.

**Related research article** Smith SJ, Davidson LA, Rebeiz M. 2020. Evolutionary expansion of apical extracellular matrix is required for the elongation of cells in a novel structure. *eLife* **9**:e55965. doi: 10.7554/eLife.55965

A key question in modern evolutionary developmental biology (evo-devo) is how new forms and structures evolve during organism development, especially those not derived from pre-existing ones. New structures can arise through various mechanisms, including gene co-option (using a pre-existing gene for a new purpose), or the acquisition of new genes (Carroll, 2008). However, to date evo-devo studies have tended to focus on the genetic regulation of these processes, and have paid relatively less attention to the cellular and extracellular processes that physically shape these new forms and structures during development.

The posterior lobe of some species of *Drosophila* fruit flies – a wedge-shaped outgrowth of the male genitalia that has a role in mating – is an example of a new structure that has evolved recently (Figure 1; Frazee and Masly, 2015). The lobe originates from a tissue called the lateral plate and is next to the claspers (a structure that helps males attach to females during mating). The lobe, which comes in various shapes and sizes, is only found in some species of the *melanogaster* subgroup of flies, including *D. melanogaster and D. sechellia* (Yassin and Orgogozo, 2013). The evolution of the lobe was made possible by co-opting the gene network that controls the development of a respiratory opening called the larval spiracle into genital development (Glassford et al., 2015). However, several questions remain unanswered: how does the posterior lobe take shape during development? And how does the development of genitalia in species with a lobe differ from that in species that do not have a lobe? Now, in eLife, Sarah Jacquelyn Smith, Lance Davidson and Mark Rebeiz from the University of Pittsburgh report the results of experiments on four different species of flies – two with lobes and two without – that shed light on these questions (Smith et al., 2020).

**Figure 1.**
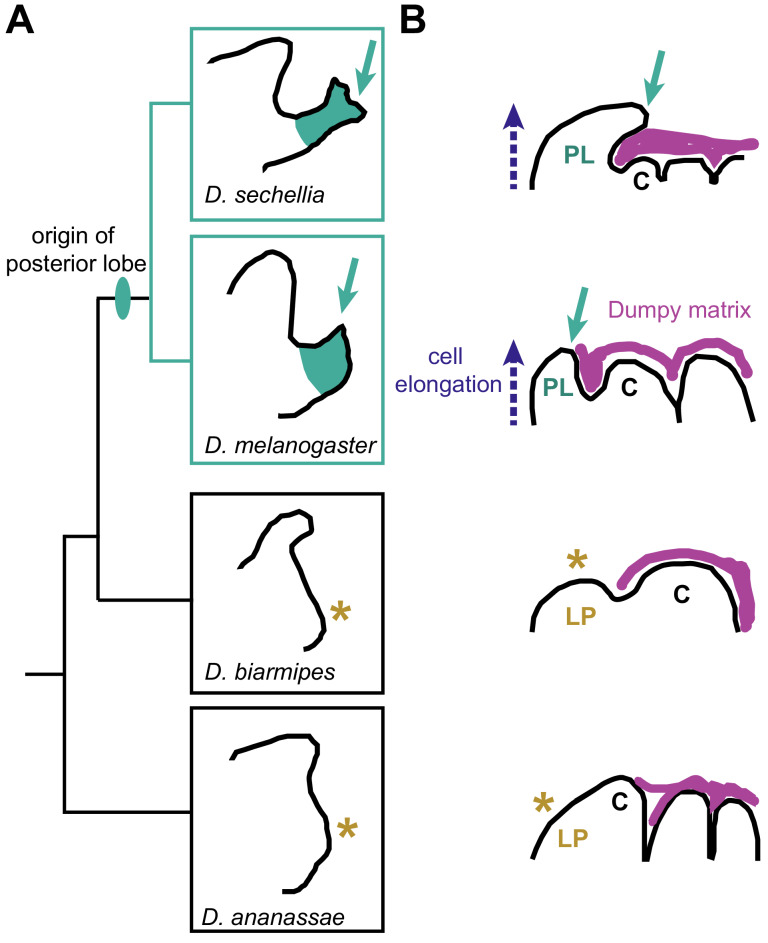
The emergence of the posterior lobe in some species of *Drosophila*. (**A**) Phylogenetic tree showing the relationship between four *Drosophila* species. The posterior lobe is a projection (indicated by a teal arrow) from the lateral plate that first appeared in the common ancestor of *D. sechellia* and *D. melanogaster.* The lobe is not found in *D. biarmipes or D. ananassae*; asterisk indicates the position on the lateral plate corresponding to the position of the lobe in the other two species. (**B**) In *D. sechellia* and *D. melanogaster* the extracellular matrix (pink) covers the entire apical side (the outer side) of the genitalia, including the elongating posterior lobe (PL) and clasper (C). In *D. biarmipes or D. ananassae* the matrix does not reach the region corresponding to the lateral plate (LP: asterisk).

Smith et al. started by asking which cellular processes underlie the formation of the posterior lobe (Heisenberg and Bellaïche, 2013). The processes they looked at included cell proliferation, polarized cell intercalation (in which cells from several layers of a tissue 'morph' into a single layer to make the tissue longer and thinner), and apical cell constriction (which involves a cell becoming thinner at one end to give it a wedge-like shape). Surprisingly, none of these processes appear to be major drivers of lobe formation. Instead, the posterior lobe in *D. melanogaster* extends from the lateral plate through a dramatic increase in cell height. Since the extracellular matrix has been known to shape organs and body parts (Brown, 2011; Tajiri, 2017), Smith et al. decided to explore if it could also drive extension in the posterior lobe.

The extracellular matrix is a mesh-like web that consists of glycoproteins, proteoglycans, and other proteins. A gigantic extracellular matrix protein called Dumpy determines the shape of tissues in *D. melanogaster* by anchoring them to the cuticle of the larva or pupa (Etournay et al., 2015; Ray et al., 2015; Wilkin et al., 2000). Smith et al. found that, in lobed species, extracellular matrix containing Dumpy covers the majority of the apical side of male genitalia including the posterior lobe as it forms (Figure 1B). In non-lobed species, however, this extracellular matrix does not cover the lateral plate. These results are consistent with the finding that lobed species express a broader pattern of Dumpy mRNA in the lateral plate and future posterior lobe. Furthermore, when Smith et al. used RNA interference to disrupt the deposition of Dumpy in the future lobe, the height of the lobe decreased in a dose-dependent manner. Thus, the amount of Dumpy in the extracellular matrix determines the shape and height of the lobe. Moreover, the extracellular matrix that associates with the lobe does not appear to directly interact with the cuticle, suggesting a new role for Dumpy during lobe formation that is independent from anchoring to the cuticle.

Several questions remain about the development and evolution of the posterior lobe. First, if Dumpy is not anchoring the lobe to the cuticle, how does the extracellular matrix containing Dumpy promote the formation of the posterior lobe? Smith et al. provide three likely scenarios: (i) Dumpy provides a structural support that helps the lobe cells elongate; (ii) the extracellular matrix mechanically pulls the lobe cells; or (iii) the extracellular matrix alters cell signaling activity during lobe development. Secondly, it is unknown whether Dumpy deposition determines the widely different posterior lobe shapes found in closely related species of the *melanogaster* group (Figure 1A). It is also unknown whether the apical extracellular matrix, via Dumpy or other matrix proteins, helps form the rudimentary lobe-like structures found in sister species such as *D. yakuba* and others (Glassford et al., 2015; Yassin and Orgogozo, 2013).

The finding that changes in the extracellular matrix can influence the evolution of the shape of tissues and organs has wide implications in evolutionary biology. Since this role for the extracellular matrix is one of the last steps in organ formation, ‘tweaking’ this step likely has an immediate effect on organ and tissue shapes without affecting the viability of organisms. Therefore, this step may be a hotspot for the evolution of cell and tissue shapes, including the evolution of new structures. The results of Smith et al. suggest new ways in which the extracellular matrix and other extracellular factors may have evolved to sculpt various new structures during development.
